# Supplementary material for: Complexome profiling of the Chlamydomonas psb28 mutant reveals TEF5 as an early PSII assembly factor
Source: Plant Cell. 2025 Mar 17;37(6):koaf055. doi: 10.1093/plcell/koaf055 (PMC12164590; doi:10.1093/plcell/koaf055)
Supplement: koaf055_Supplementary_Data [file koaf055_supplementary_data.zip › psb28_interactive.zip!/plots/intensity/qualityPlots/peptides/PRK1_Cre12.g554800.t1.2.html]

### Cre12.g554800.t1.2

Trivial name: PRK1  
SeqCoverage [median]: 5.10  
Mass: 41.89 kDa  
  
 

| Sequence | MissedCleavage | Mass [Da] | wtIntensity | muIntensity | Score |
| --- | --- | --- | --- | --- | --- |
| FYGEITQQMLK | 0 | 1356.67 | 363.43 | 2285.79 | 112.11 |
| VAELLDFK | 0 | 933.52 | 0.00 | 1583.08 | 75.85 |
|  |  |  | 363.4 | 3868.9 |  |
